# Cyclin-dependent kinase inhibitors in malignant hematopoiesis

**DOI:** 10.3389/fonc.2022.916682

**Published:** 2022-08-11

**Authors:** Alessia Schirripa, Veronika Sexl, Karoline Kollmann

**Affiliations:** Institute of Pharmacology and Toxicology, University of Veterinary Medicine Vienna, Vienna, Austria

**Keywords:** cyclin-dependent kinase inhibitors, hematopoiesis, hematopoietic diseases, INK4 family, Cip/Kip family

## Abstract

The cell-cycle is a tightly orchestrated process where sequential steps guarantee cellular growth linked to a correct DNA replication. The entire cell division is controlled by cyclin-dependent kinases (CDKs). CDK activation is balanced by the activating cyclins and CDK inhibitors whose correct expression, accumulation and degradation schedule the time-flow through the cell cycle phases. Dysregulation of the cell cycle regulatory proteins causes the loss of a controlled cell division and is inevitably linked to neoplastic transformation. Due to their function as cell-cycle brakes, CDK inhibitors are considered as tumor suppressors. The CDK inhibitors p16^INK4a^ and p15^INK4b^ are among the most frequently altered genes in cancer, including hematopoietic malignancies. Aberrant cell cycle regulation in hematopoietic stem cells (HSCs) bears severe consequences on hematopoiesis and provokes hematological disorders with a broad array of symptoms. In this review, we focus on the importance and prevalence of deregulated CDK inhibitors in hematological malignancies.

## 1 Introduction

Cell-cycle progression is a fundamental biological process which requires tight regulation to guarantee a correct cell division. Perturbations of cell cycle components may provoke an uncontrolled cell proliferation. Dysregulated G1-S transition is a common feature of tumor development and associated with genetic alterations of key regulators of the cell-cycle machinery (1). Based on their function as a cell cycle brake, CDK inhibitors (CKIs) mainly act as tumor suppressors and are frequently deactivated in human neoplasia (2–4).

## 2 CKIs regulate the cell cycle

**Figure 1:**
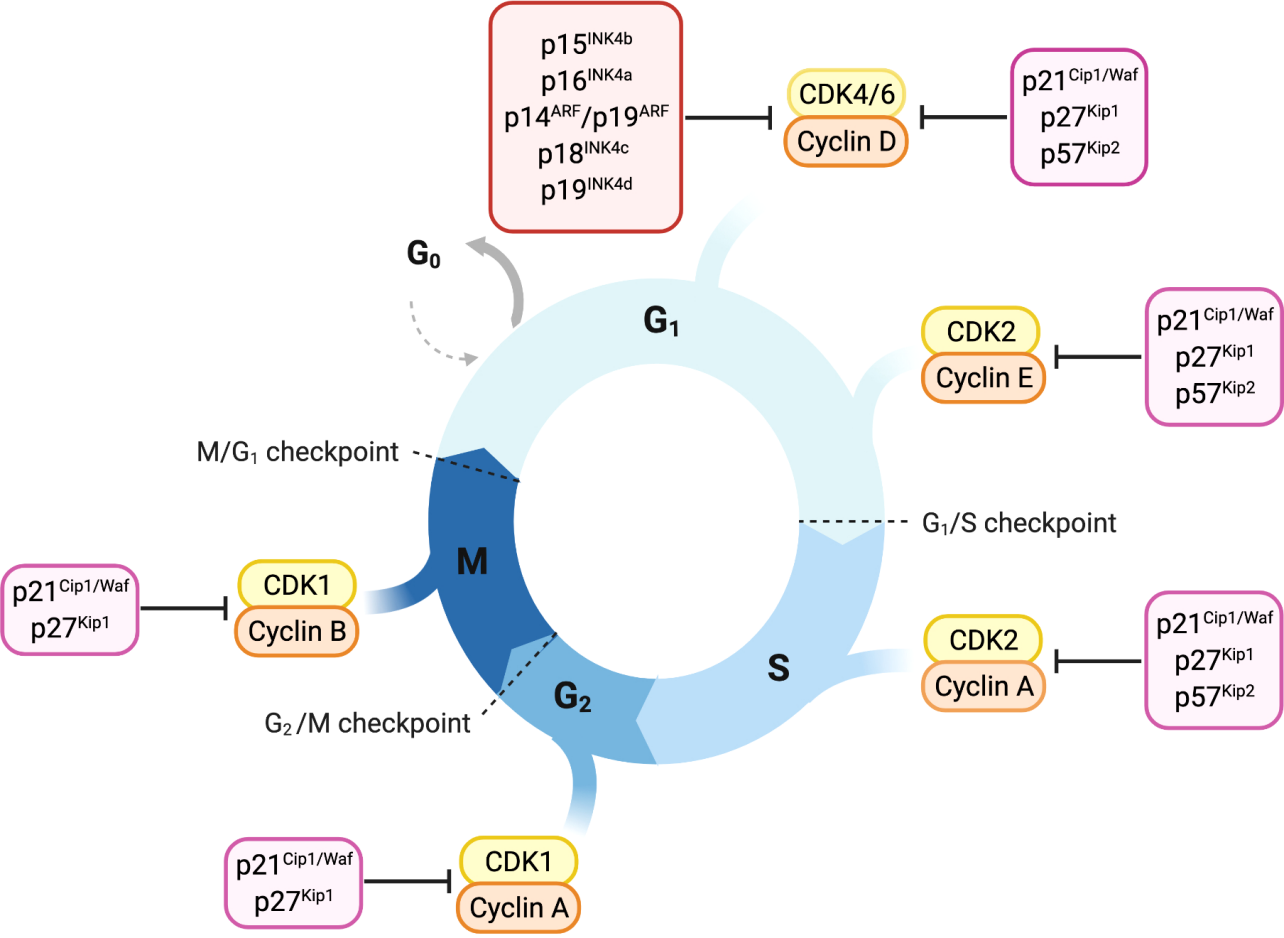
Overview of cell-cycle control and its main regulators. Progression through cell cycle phases is governed by different CDK-cyclin complexes and the respective cyclin-dependent kinase inhibitors. Members of the INK4 family, p16^INK4a^, p15^INK4b^, p18^INK4c^ and p19^INK4d^, specifically bind and inhibit CDK4/6-cyclin D complexes promoting cell cycle arrest in the G1 phase. The Cip/Kip proteins including p21^Cip1/Waf^, p27^kip1^ and p57^Kip2^, play their role as cell-cycle inhibitors by counteracting a broader spectrum of CDK-cyclin complexes. p21^Cip1/Waf^, p27^kip1^ and p57^Kip2^ restrain cell-cycle both during early and late G1 phase by binding either CDK4/6-cyclin D or CDK2-cyclin E complexes. Later in the cell-cycle, they can bind and inhibit CDK2-cyclin A complex, thus imposing a brake during the S-phase. p21^Cip1/Waf^ and p27^kip1^ are able to delay entry in the M phase by inhibiting CDK1-cyclin A complex and thereby prevent the progression through mitosis counteracting CDK1-cyclin B complex.

Cyclin-dependent kinases (CDKs), their activating cyclins and CDK inhibitors guide cells through the cell cycle (**Figure 1**). Distinct cyclins are periodically produced and assemble to cyclin-CDK complexes that drive the specific cell-cycle steps, from G1 to M phase. Fine tuning is achieved by inhibitory phosphorylation or binding of CDK inhibitory subunits (CKls) (5–7).

Cyclin-dependent kinase 4 (CDK4) and CDK6 are closely related serine/threonine kinases responsible for driving cells through the G1 phase. Mitogenic signals induce transcription of D-type cyclins (D1, D2 and D3). Their association with CDK4 and CDK6 leads to kinase activation and phosphorylation of the retinoblastoma protein (Rb) (8). CDK-dependent Rb phosphorylation releases Rb from E2F transcription factors and induces transcription of E2F target genes required for S-phase entry (9). G1-S transition is then initiated by CDK2-cyclin E/A complexes, which are active during the entire S-phase (10–12). CDK1 activity is low during G1/S transition but raises during G2-M phase, controlling the initiation of mitosis (13, 14).

CDK-cyclin activity is counterbalanced by members of the two CDK inhibitor families, the INK4 family and the Cip/Kip family (8). p16^INK4a^, p15^INK4b^, p18^INK4c^ and p19^INK4d^ are the members of the INK4 family and are specific for CDK4 and CDK6 (15). In response to anti-proliferative signals, INK4 proteins are transcribed and bind CDK4 and CDK6 causing a conformational change which reduces their affinity for D-type cyclins (16).

The Cip/Kip family consists of p21^Cip1/Waf^, p27^Kip1^ and p57^Kip2^. In contrast to INK4 proteins,

Cip/Kip proteins have the ability to bind CDK4/6-cyclin D and CDK-cyclin A/B/E complexes (8, 16–19). p21^Cip1/Waf^ and p27^Kip1^ are described to have a dual function in cell cycle regulation. Whereas they mainly inhibit CDK-cyclin activity they have been reported to also enhance the assembly of CDK4/6-cyclin D complexes, resulting in a proliferative advantage for the cell (18, 20, 21).

When present at low levels, p21^Cip1/Waf^ preferentially binds to CDK4/6-cyclin D complexes, facilitating complex formation, nuclear localization and cell-cycle progression. In response to DNA damage and p53 stimulation, p21^Cip1/Waf^ accumulates at high levels in a cell and provokes a robust cell cycle arrest by inhibiting CDK2- cyclin E-A complexes (8, 22–25). The mechanism behind these observations is given by *in vitro* experiments showing that changes in p21^Cip1/Waf^ stoichiometry reflect the conversion of active to inactive cyclin-CDK complexes. Active complexes contain a single p21^Cip1/Waf^ molecule, while two molecules are required for complex inhibition (26, 27).

This double-faced role has been described also for p27^Kip1^. On the one hand, p27^Kip1^ binds to the conserved cyclin box residues thus promoting the subsequent complex formation between p27^Kip1^-cyclin A and CDK2. Upon complex formation, p27^Kip1^ induces a distortion on the CDK2 N-terminal lobe in proximity of CDK2 catalytic site, thereby preventing ATP binding. On the other hand, phosphorylated p27 ^Kip1^ binds to CDK4 leading to a remodeling of the ATP site and results in increased RB phosphorylation. Data suggest a similar mechanism for p21^Cip1/Waf^ activating CDK4 *via* phosphorylation sites (28).

p57^Kip2^ mainly functions during G1-S and G2-M transitions where it blocks any CDK-cyclin complexes. No cell cycle activating mechanisms have been described yet.

The Cip/Kip members, p57^Kip2^ and p21^Cip1/Waf^ are major players in cellular stress responses, where they balance the induction of cell cycle arrest, apoptosis and senescence (29). p21^Cip1/Waf^ has a unique role as it mediates cell cycle arrest downstream of the tumor suppressor p53 (22). A variety of cellular stresses, such as DNA damage and oncogene activation, stimulate p53 expression, which in turn transactivates its targets including the pro-apoptotic genes Bax, PUMA and Noxa as well as p21^Cip1/Waf^ (30–32). Therefore, p21^Cip1/Waf^ might be an exploitable candidate for therapeutic intervention in p53 mutated tumors.

## 3 CKIs in hematopoietic stem cells

Under homeostatic conditions, hematopoietic stem cells (HSCs) reside in the hypoxic bone marrow niche in a quiescent state (33–35). When needed, HSCs rapidly enter the cell cycle to replenish peripheral hematopoiesis. Self-renewal and differentiation are tightly balanced to maintain the stem cell pool while giving rise to hematopoietic progenitors, which ultimately differentiate into mature blood cells (35, 36). The delicate balance between quiescence and proliferation in HSCs requires a strictly controlled cell cycle progression.

Cyclin dependent kinase inhibitors (CKIs) represent a major break for cell cycle entry and the prevention of uncontrolled proliferation. Several studies started to unravel the impact of CKIs in HSCs (37–40).

**Figure 2:**
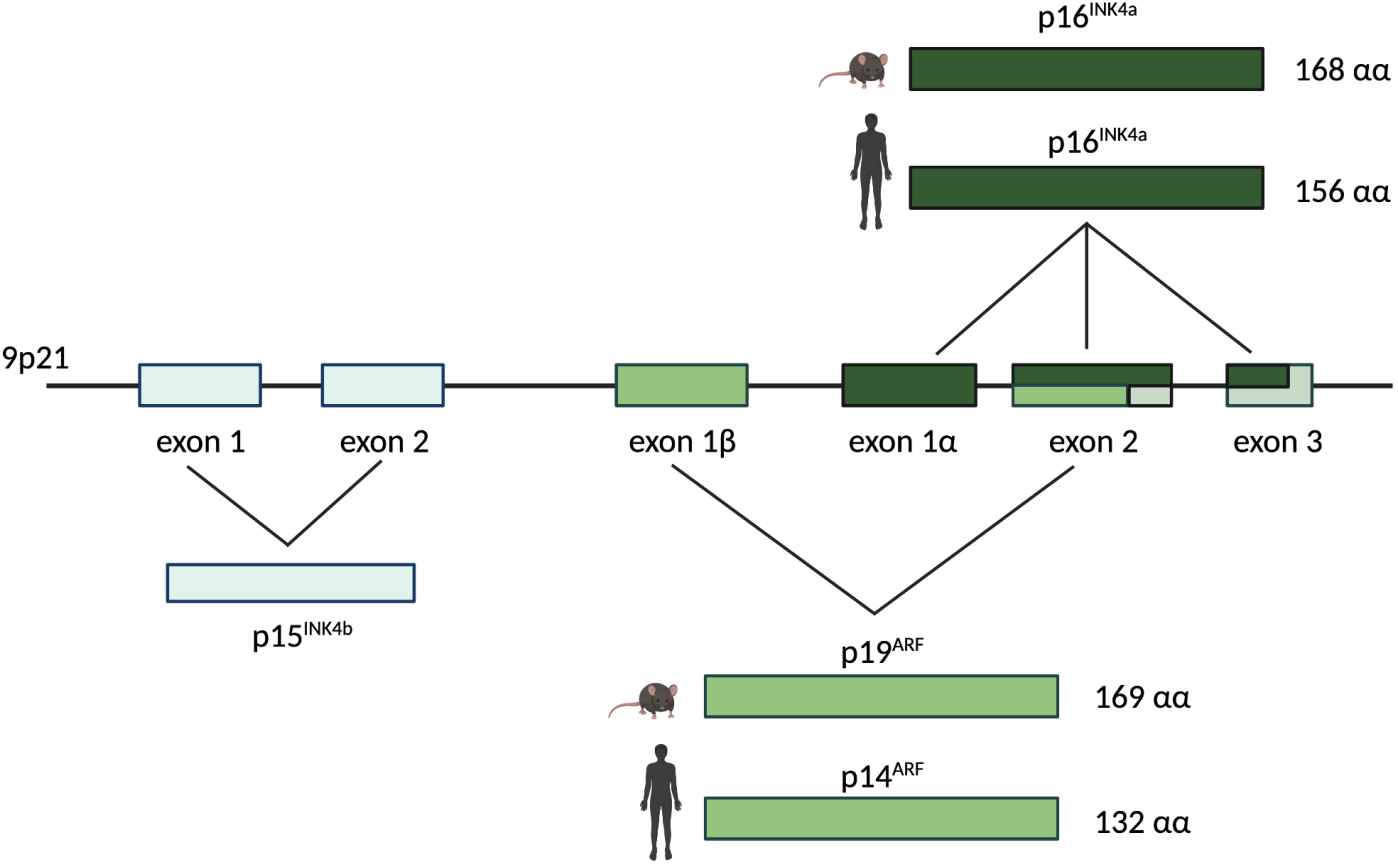
The human/murine *INK4a/ARF* locus. The *INK4a/ARF* locus resides on chromosome 9p21 and encodes for two different proteins in human and mouse: p16^INK4a^ and p14^ARF^ (named p19^ARF^ in mouse). The *INK4a* gene is represented by exons 1α, 2, and 3 and it encodes for p16^INK4a^, a 168 amino acids protein in mouse and a 156 amino acids protein in human. The *ARF* gene is composed by exons 1β, 2, and 3. It encodes for p19^ARF^ in mouse (169 amino acids) and for p14^ARF^ in human (132 amino acids). Upstream of the *INK4a* and *ARF* genes on the same chromosome, exons 1 and 2 represent the *INK4b* gene encoding for p15^INK4b^.

p16^INK4a^ is encoded by exons 1α, 2 and 3 of the *INK4a* locus (**Figure 2**). A different transcript derived from the same locus, encoded by the exons 1β, 2 and 3, encodes for the protein p19ARF (**Figure 2**) which has the capacity to block the cell cycle progression at the G1 and G2 phase (41–43). Thus, the INK4a locus represents a master growth regulator through its capacity to interface with both proliferation (Rb pathway *via* p16INK4a) and apoptosis (p53 pathway *via* p19ARF) (4, 44).

The transcriptional repressor Bmi-1 is part of the Polycomb group and it is present at high levels in HSCs (45–47). Bmi-1 represses the *INK4a* locus, thus limiting *p16^INK4a^* and *p19^ARF^* expression (39, 48). Bmi-1 deficiency impairs HSCs self-renewal as it increases p16^INK4a^ and p19^ARF^ levels thereby leading to proliferative arrest and cell death (39). Mice lacking *p16^INK4a^* do not show any dramatic effect on hematopoiesis, which could be explained by the reported low *p16^INK4a^* expression in normal HSCs (49, 50).

*p16^INK4a^* expression increases in HSCs with aging and this is associated with lower HSC numbers. p16^INK4a^ inhibition counteracts the reduced HSC maintenance associated with aging, improves their repopulation ability and mitigates apoptosis (51).

The role of p16^INK4a^ and p19^ARF^ for the regulation of hematopoietic progenitor cells becomes evident in mice harboring a targeted deletion of the *INK4a* locus that eliminates both proteins. Young *p16^INK4a-/-^/p19^ARF-/-^* mice show extramedullary hematopoiesis in the spleen with a high proportion of lymphoblasts and megakaryocytes in the red pulp and proliferative expansion of the white pulp. Aging aggravates this phenomenon and extends extramedullary hematopoiesis to nonlymphoid organs (49).

Among the CKIs, p18^INK4c^ is the most powerful player and cell cycle inhibitor involved in murine HSC self-renewal (40, 52). *p18^INK4c^* deficient mice show HSCs with enhanced self-renewal ability which leads to the expansion of the HSC pool. This is also evident in serial transplantation experiments where *p18^INK4c^* deletion allows for an advanced HSC repopulation ability (40, 53).

Information on p15^INK4b^ and p19^INK4d^ in regulating HSC function is scarce. Characterization of the hematopoietic stem and progenitor cells of *p15^INK4b^* deficient mice revealed an increased frequency in common myeloid progenitors, but no alterations in the HSC compartment (54, 55).

The need to get first insights into the role of p19^INK4d^ in HSCs leads to the characterization of the hematopoietic system of mice lacking *p19^INK4d^*. Knockout mice do not reveal any defect under homeostatic conditions (56). However, *in vitro* studies highlight the involvement of p19^INK4d^ in megakaryopoiesis, where it regulates the endomitotic cell cycle arrest coupled to terminal differentiation (57).

Moreover, p19^INK4d^ effects become evident when HSCs are exposed to genotoxic stress. In this context, p19^INK4d^ is required to maintain HSCs in a quiescent state, protecting them from apoptosis as genotoxic substances act during the S-phase (58).

The p53 induced CKI p21^Cip1/Waf^ also regulates effects upon stress. Bone marrow transplantation experiments, using cells derived from mice after 2 Gy irradiation show that *p21^Cip1/Waf^* deficiency leads to a significantly reduced repopulation ability (37, 59).

In contrast, *p27^Kip1^* knock-out mice lack any perturbations in HSC number, self – renewal ability or cell-cycle state. The role of p27^Kip1^ is restricted to more committed progenitor cells where its deletion increases proliferation and the pool size of Sca1^+^Lin^+^ cells (38).

In quiescent HSCs p57^Kip2^ dominates as major CKI, where it is expressed at high levels. *p57^Kip2^* deficiency reduces the HSC population, compromises the maintenance of quiescence and impairs repopulation capacity (60).

In summary this led us to conclude that CKIs have distinct essential roles in hematopoietic stem and progenitor cells that are only partially understood. Whereas Cip/Kip proteins are predominantly involved in stress responses, INK proteins dominate in the control of hemostatic conditions.

## 4 Alterations in CKIs

In human cancers the *INK4a-ARF-INK4b* locus at chromosome 9p21 is one of the most frequently mutated and epigenetically silenced sites (61–63). This locus encodes for the cyclin dependent kinase inhibitors p16INK4a and p15INK4b and for the tumor suppressor protein p14^ARF^ (p19^ARF^ in the mouse), which is induced upon p53 activation (**Figure 2**) (64, 65). Many solid tumors including melanoma, pancreatic adenocarcinomas, esophageal and non-small cell lung carcinoma, harbor mutations in the *p16^INK4a^* and *p15^INK4b^* genes. In hematological malignancies *p16^INK4a^* and *p15^INK4b^* are frequently deleted e.g. in chronic myeloid leukemia (CML) and acute lymphoblastic leukemia (ALL) (66–70).

*p18^INK4c^* and *p19^INK4d^*, mapped on chromosome 1p32 and 19p13.2 respectively (71, 72), are involved in the development of a more distinct set of tumors. Somatic mutations of *p18^INK4c^* are associated with medullary thyroid carcinoma, hepatocellular carcinoma and breast cancer (73–75). Only little information is available regarding the role of p19^INK4d^ in human malignancies; frame shift mutations and rearrangements in the *p19^INK4d^* gene have been documented in osteosarcoma (76), while its loss or downregulation have been detected in hepatocellular carcinoma (77) and testicular germ cell tumors (78).

The deletion of the Cip/Kip proteins in mice leads to an increased development of malignancies (79–81), underlining their main role as tumor suppressors. Contradictorily, in some tumor types Cip/Kip proteins also display an oncogenic activity when relocated to the cytoplasm (82–84).

Low p27^Kip1^ levels are associated with more aggressiveness and poor prognosis in several human cancers (85–87). Control of p27^Kip1^ levels involves a nuclear to cytoplasmic redistribution which is regulated by phosphorylation sites on distinct residues. Mitogenic signals induce p27^Kip1^ phosphorylation on Ser10, inducing nuclear export (88, 89), while phosphorylation on Thr198, mediated by PKB/Akt, promotes p27^Kip1^ association with 14-3-3 proteins and its transport to the cytoplasm (90).

Whereas nuclear p27^Kip1^ inhibits cell proliferation and suppresses tumor formation, cytoplasmatic p27^Kip1^ is involved in cytoskeleton rearrangement and contributes to cell migration (82, 89) and may promote metastasis. In some hematologic malignancies (91–93) and carcinomas (such as breast, esophagus, cervix and uterus tumors) (94–98), a positive association of cytoplasmic p27^Kip1^ levels with a poor clinical outcome has been reported.

p21^Cip1/Waf^ acts as a tumor suppressor in breast, colorectal, gastric, ovarian and oral cancers. Similar to p27^Kip1^ it may display oncogenic activities when retained in the cytoplasm. p21^Cip1/Waf^ cytoplasmic accumulation is caused by phosphorylation at Thr145 by activated AKT1 (99). Through the association with proteins involved in the apoptotic process, cytoplasmatic p21^Cip1/Waf^ mediates their inhibition, thus exhibiting anti-apoptotic effects. As such, cytoplasmic p21^Cip1/Waf^ is indicative for aggressiveness and poor survival in prostate, cervical, breast and squamous cell carcinomas (100).

In contrast, the role of p57^Kip2^ is limited at being a tumor suppressor, as there is so far no evidence of an oncogenic role so far (101–104).

Given the extensive knowledge regarding the role of CDK inhibitors in tumor biology there is increasing interest in exploiting them as potential target for cancer treatments. Here we review and discuss the importance they play in hematopoietic malignancies.

## 5 CKIs in hematologic malignancies

**Figure 3:**
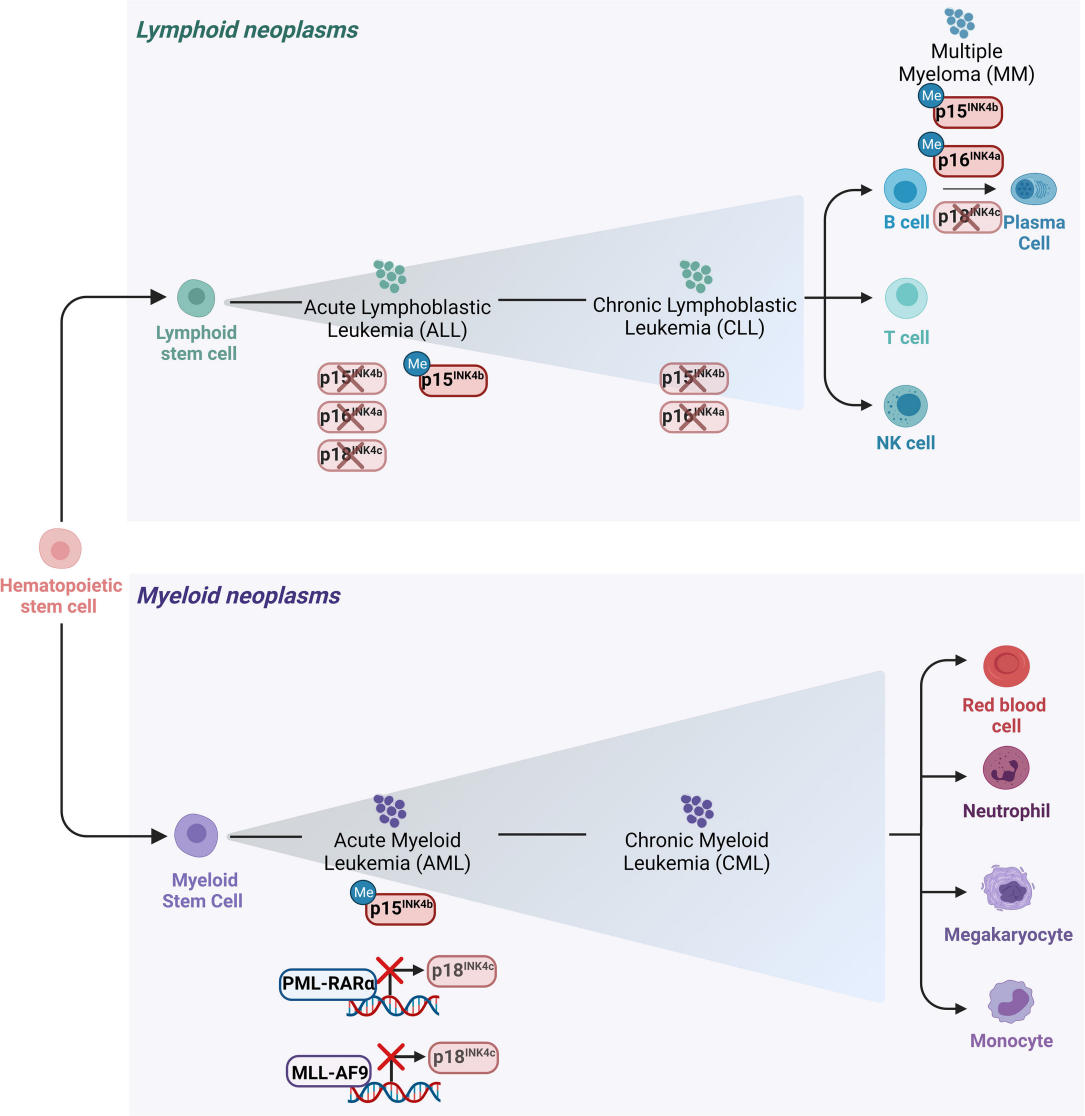
Main alterations of the INK4 proteins in leukemia and lymphomas. Schematic representation of the hematopoietic tree and main alterations affecting the INK4 proteins in different hematopoietic malignancies. Deletion of *p15^INK4b^* and *p16^INK4a^* together with their 5’ CpG islands hypermethylation in their promoter regions are the most frequent modes of *p15^INK4b^* and of *p16^INK4a^* inactivation in various subtypes of hematopoietic neoplasms including ALL and CLL. Deletion of *p18^INK4c^* has been rarely observed in ALL, whereas it is frequently deleted in MM. *p18^INK4c^* is subjected to a transcriptional repression imposed by the oncofusion protein PML-RARα in APL blasts and it is similarly downregulated by MLL-AF9 in cell lines derived from AML patients.

**Figure 4:**
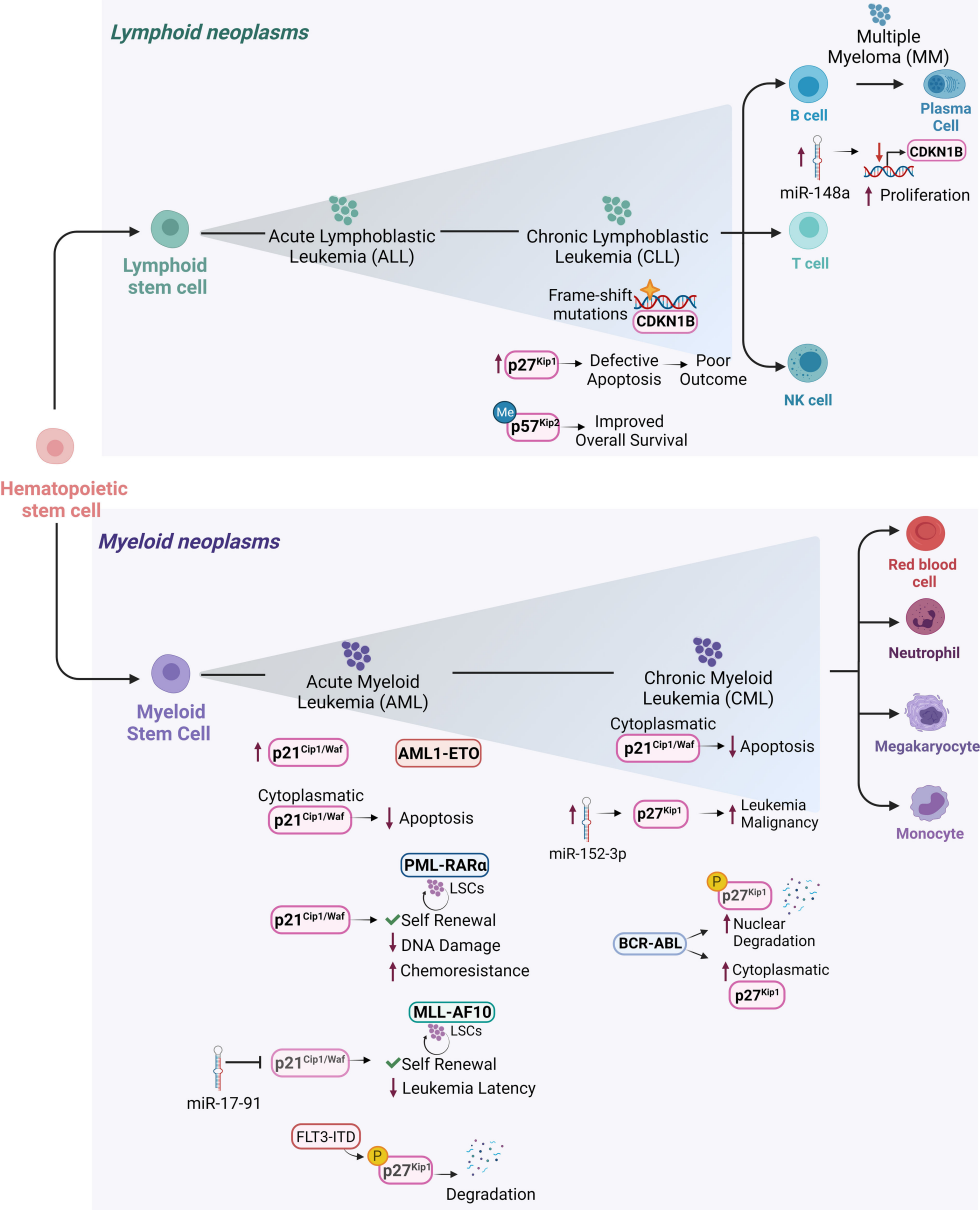
Cip/Kip proteins main deregulations and functions in different hematopoietic malignancies. Schematic representation of the hematopoietic tree and main functions exerted by Cip/Kip proteins in different hematopoietic malignancies. Increased p21^Cip1/Waf^ levels have been reported in AML1-ETO positive AML patients, where it is believed to support LSCs maintenance and self-renewal ability. p21^Cip1/Waf^ anti-apoptotic functions associated with its cytoplasmatic localization have been observed in AML blasts and in cell lines derived from human CML in blast crisis. In PML-RARα LSCs, *p21^Cip1/Waf^* expression maintains self-renewal of LSCs and limits DNA damage, thus protecting them from functional exhaustion and conferring chemoresistance. In MLL-AF10 induced AML, *p21^Cip1/Waf^* suppression mediated by miR-17-91 leads to decreased leukemia latency. Elevated p27^Kip1^ levels in B-CLL where they confer protection against apoptosis, are associated with poor outcome. In hairy cell leukemia, a form of B-CLL, *CDKN1B* gene encoding for p27^Kip1^ is the second most common altered gene by frame shift mutations. In MM, higher miR-148a levels correlate with decreased *CDKN1B* expression leading to sustained proliferation. In CML, overexpression of miR-152-3p targets p27^Kip1^ and promotes leukemia malignancy. In AML, p27^Kip1^ is subjected to FLT3-ITD phosphorylation (pY88- p27Kip1) which mediates p27^Kip1^ degradation. BCR-ABL1^+^ CML can promote degradation of nuclear p27^Kip1^ and to increased cytoplasmatic p27^Kip1^, thus compromising p27^Kip1^ tumor suppressor activity and promoting leukemic cell survival. *p57^Kip2^* gene has been frequently found methylated in diffuse large B-cell lymphoma patients, where the low-risk group it is associated with a more favorable overall survival.

Hematologic malignancies consist of a spectrum of malignant neoplasms that affect bone marrow, blood and lymph nodes and originate from the uncontrolled proliferation of hematopoietic cells. They are driven by genetic and epigenetic aberrations, which can be exploited for diagnosis and therapeutic decisions. The dominant alterations of CKIs are reviewed below and illustrated in **Figures 3**, **4**.

### 5.1 INK4 proteins in leukemia and lymphoma

#### 5.1.1 p16^INK4a^ and p15^INK4b^

The *CDKN2A/B* locus encodes for p16^INK4a^, p14^ARF^ (p19^ARF^ in mice) and p15^INK4b^. This locus is affected by deletion, mutation or promoter hyper-methylation (62, 63) and frequently altered in patients with hematologic malignancies (4, 105, 106). The design of mouse strains with single or multiple targeted disruptions of the *p16^INK4a^*, *p19^ARF^* and *p15^INK4b^* loci shed light on their distinct roles.

*p19^ARF-/-^* mice spontaneously develop a variety of tumors already by the age of 2 months. Analysis of diseased mice shows that T cell lymphoma is the second most common tumor type (107, 108). In line, *p19^ARF-/-^* newborn mice exposed either to X-ray or to γ-irradiation develop anaplastic T cell lymphoma (107, 108). In an acute lymphoblastic leukemia (ALL) model, the loss of *p19^ARF^* initiates a more aggressive disease BCR-ABL1+ transformation. In this model, *p19^ARF^* deletion also confers resistance to the kinase inhibitor imatinib (109). These data suggest a specific role for p19^ARF^ in the lymphoid lineage. Therefore, it would be interesting to analyze if p19^ARF^ could serve as a marker for prognosis and therapeutic outcome.

Homozygous deletion of p16^INK4a^ is not associated with an increased spontaneous cancer development. Of note, the concomitant heterozygous loss of *p19^ARF^* in *p16^INK4a-/-^* animals increases tumorigenesis and provokes the development of a wide spectrum of malignancies, including lymphoma (110). Importantly, the spontaneous tumors originating from mice harboring the heterozygous loss of *p19^ARF^* and *p16^INK4a^* homozygous deletion, retain the second *p19^ARF^* allele. However, the observed increased tumorigenesis in *p16^INK4a-/-^* mice upon heterozygous *p19^ARF^* loss underlines the cooperation of the two tumor suppressors.

Young mice show spontaneous tumorigenesis and a higher sensitivity to carcinogenic treatments, especially B cell lymphoma (49).

*p15^INK4b-/-^* mice show lymphoproliferative disorders including lymphoid hyperplasia in the spleen and formation of secondary follicles in lymph nodes but rarely develop lymphoma. This suggests that p15^INK4b^ controls homeostasis of the hematopoietic compartment, rather than acting as a tumor suppressor (111).

Although p15^INK4b^ and p16^INK4a^ function as repressors of the cell cycle, in view of the phenotypes shown by the mouse models described above, they seem to have roles in different contexts. p15^INK4b^ is mainly responsible for homeostasis and p16^INK4a^, together with p19^ARF^, is more involved in regulating the response to oncogenic stress. This suggests that p16^INK4a^ might function as a sensor of oncogenic signals thus representing a safeguard against neoplasia.

*CDK4^R24C^/CDK6^R31C^* double knock-in mice have been used to address the importance of INK4 inhibitors in regulating CDK4 and CDK6. INK4 binding is prevented by introducing point mutations in CDK4 (R24C) and CDK6 (R31C). The CDK4^R24C^ mutation has been initially identified in hereditary melanoma and shows elevated CDK4 kinase activity (112). So far the *CDK6^R31C^* mutation has not been found in patients but is used to investigate CDK6-INK4 interactions. *CDK4^R24C^/CDK6^R31C^* mice show a shortened survival caused by the onset of primary endocrine epithelial or hematopoietic malignancies. Mice injected with CDK4^R24C^/CDK6^R31C^ BCR-ABL1 transformed cell lines display accelerated tumor growth and reduced disease latency (113). This analysis highlights the crucial importance of INK4 binding to control CDK4/CDK6 activity in hematopoiesis. Therefore, it is attractive to conclude that CDK4/6 inhibitors are effective in patients that lack appropriate INK4-mediated control.

First evidence indicated that the *CDKN2* locus in human tumor cell lines derived from solid tumors is predominantly homozygously deleted and thereby *p16^INK4a^* becomes inactivated. This was later verified also for leukemia and lymphoma; only a low frequency of point mutations has so far been documented (114–118).

Studies in primary leukemia also identified alterations in *p15^INK4b^*. The highest frequency of homozygous deletions of *p16^INK4a^* or *p15^INK4b^* occurs in ALL, while they are heterozygously deleted in chronic lymphocytic leukemia (CLL) (114, 119–121). T-ALL is most frequently associated with *p16^INK4a^* loss, while *p15^INK4b^* deletions are more often observed in pediatric ALL (70, 106, 119, 122–127). Initial studies focused their attention on the frequency of *p16^INK4a^* and *p15^INK4b^* mutations in adult and childhood ALL (70, 114, 120, 122, 128). Only at later stages the potential of these genes as prognostic factors was taken into account.

The overall incidence of *p16^INK4a^* deletion is higher than *p15^INK4b^*. Patients with *p15^INK4b^* deletions harbor *p16^INK4a^* co-deletions, which is not consistently observed vice versa. Cases with homozygous *p16^INK4a^* deletion either maintain an unmutated *p15^INK4b^* gene or show a hemizygous *p15^INK4b^* deletion. These findings point at *p16^INK4a^* as the central target of deletions which play the central role for ALL leukemogenesis (70, 119, 120, 123).

The prognostic significance of *p16^INK4a^* and *p15^INK4b^* deletions remains a matter of debate with contradictory reports: some studies showed an adverse prognostic effect (122, 123, 127, 129–133), which was not confirmed by others (70, 134–136).

Analysis of mixed leukemia types, small patient cohorts or insensitive molecular techniques, like polymerase chain reaction (PCR), immunocytochemistry and fluorescence *in situ* hybridization (FISH) may have complicated the interpretation. The conclusion of some studies still leaves the potential implication of *p16^INK4a^* and *p15^INK4b^* deletions in patient prognosis elusive.

Point mutations in the *CDKN2A/CDKN2B* genes, encoding for p16^INK4a^ and p15^INK4b^ respectively, are sporadically found in human hematopoietic disorders. A comprehensive analysis of 264 T-ALL cases, searching for mutations in cell cycle genes, found *CDKN2A/CDKN2B* as the most mutated ones (137). Inactivation of *p15^INK4b^* and *p16^INK4a^* genes can also be based on hypermethylation of the 5’ CpG islands in their promoter regions which induces transcriptional silencing (138). This mode of *p16^INK4a^* inactivation is commonly found in breast and colon cancer (139) but also in leukemia and lymphoma. Normal hematopoietic cells lack *p15^INK4b^* and *p16^INK4a^* promoter hypermethylation, which only occurs *de novo* upon malignant transformation (140). Interestingly, *p15^INK4b^* or *p16^INK4a^* seem unaffected at any stage of CML (140), whereas hypermethylation of *p15^INK4b^* and *p16^INK4a^* is a common event in multiple myeloma (MM) (141). Selective *p15^INK4b^* promoter hypermethylation, without *p16^INK4a^* alterations, is observed in acute myeloid leukemia (AML), myelodysplastic syndrome and ALL (140, 142–146), whereas Burkitt’s lymphoma and Hodgkin’s lymphoma present p16INK4a hypermethylation (140, 141, 147–150).

Overall, the current available data show that inactivation of *p15^INK4b^* and *p16^INK4a^* in human hematopoietic malignancies is caused by genetic deletion or promoter hypermethylation. Linking these alterations in a well-evaluated cohort of patients would be extremely precious to finally define their role for disease progression and their prognostic relevance. The frequency of their alterations in leukemia and lymphoma is indicative of a central role and renders them promising candidates for novel therapeutic approaches.

#### 5.1.2 p18^INK4c^

Being the functionally most relevant INK in HSC regulation under stress conditions, it is not surprising that the absence of p18^INK4c^ provokes hematopoietic abnormalities and extramedullary hematopoiesis (111). Mice lacking *p18^INK4c^* experience the consequences of the absence of its tumor suppressor function and its role in controlling lymphocyte homeostasis (111, 151). *p18^INK4c-/-^* mice spontaneously develop neoplasia including angiosarcoma, testicular tumors, pituitary tumors and lymphoma.

*p18^INK4c^* mutations in human hematopoietic malignancies are surprisingly rare in acute leukemias, as they have not been identified in AML and deletions have been reported in just some cases of adult ALL (70, 152, 153). *p18^INK4c^* maps on the chromosomal region 1p32. In line with data showing no involvement of p18^INK4c^ in childhood AML (70), no alterations of the 1p region in childhood ALL have been found so far (154). Similarly, no evidence for *p18^INK4c^* promoter hypermethylation in acute leukemia has been reported (155).

In MM, *p18^INK4c^* is frequently deleted, whereas no point mutations have been detected (156, 157).

In normal B-cells, p18^INK4c^ controls the cell cycle and is involved in the terminal differentiation of B-cells into plasma cells through the inhibition of CDK6 (158, 159). Despite that role, *p18^INK4c^* expression is preserved in most lymphoid malignancies (68, 118). The hemizygous loss of p18^INK4c^ has been reported in mantle cell lymphoma, but not in Hodgkin’s lymphoma, where *p18^INK4c^* is frequently repressed due to promoter hypermethylation (160–162).

The oncofusion protein PML-RARα which drives acute promyelocytic leukemia (APL) directly suppresses *p18^INK4c^* expression which is downregulated in APL blasts compared to normal promyelocytes (163).

ChIP-seq experiments of MLL and AF9 in THP-1 cells reveal the *CDKN2C* locus, encoding for p18^INK4c^, as a binding region. This indicates that *p18^INK4c^* expression is subject to MLL-AF9 mediated regulation (164).

A detailed map of p18^INK4c^ regulation in different leukemic subtypes is still missing and would help clarifying the role of p18^INK4c^ in hematopoietic malignancies and leukemic stem cells (LSCs). The data currently available are indicative for sporadic alterations of *p18^INK4c^* in hematologic malignancies.

#### 5.1.3 p19^INK4d^

The analysis of *p19^INK4d^* knock-out mice failed to detect any tumor suppressing effects of p19^INK4d^. Mice lacking p19^INK4d^ do not spontaneously develop tumors and no abnormalities of the hematopoietic system are evident (56). In line, alterations of *p19^INK4d^* are not general hallmarks of hematopoietic neoplasms (76, 165) albeit the data available are scarce. The absence of a mouse phenotype in terms of enhanced cell proliferation and tumor development upon *p19^INK4d^* loss suggests a functional compensation exerted by the other INK4 or Cip/Kip proteins.

### 5.2 Cip/Kip proteins in leukemia and lymphoma

#### 5.2.1 p21^Cip1/Waf^

p21^Cip1/Waf^ is a key mediator of p53-dependent tumor suppressor functions (22) and acts as a negative regulator of cell cycle progression. p21^Cip1/Waf^ and its role in cellular proliferation have been described in a vast body of literature. Its negative function on cell cycle progression indicates that p21^Cip1/Waf^ may exert tumor suppressive roles and participates in leukemia development even under wild type p53 conditions.

*p21^Cip1/Waf^* deficient mice are viable and fertile (166, 167). In those mice, harboring wild type p53, spontaneous tumor development occurs late in life at an average age of 16 months. The variety of malignancies includes tumors of hematopoietic, vascular and epithelial origin. For instance, 14% of all tumors are B-cell lymphoma (168).

The tumor spectrum developed by *p21^Cip1/Waf^* deficient mice is remarkably similar to the one observed in p53 deficient mice, which is not surprising keeping in mind the p21^Cip1/Waf^ activation by p53. However, p53 deficient mice are characterized by longer latency. However, *p21^Cip1/Waf^* deficient mice do not develop T-cell lymphoma, one of the most frequent tumors arising in p53 deficient mice.

The clinical relevance and potential as a prognostic marker of aberrant *p21^Cip1/Waf^* expression has been assessed in various types of human cancers.

Loss of p21^Cip1/Waf^ protein levels correlates with a more advanced tumor stage and worse prognosis in pancreatic cancer (169), while its overexpression has been shown to be associated with poor prognosis in non-small cell lung cancer (170) and in esophageal squamous cell carcinoma patients (171).

Interestingly, other studies report low *p21^Cip1/Waf^* expression being associated with reduced survival in patients affected by esophageal carcinoma (172, 173).

The relationship between *p21^Cip1/Waf^* expression and gastric cancer remains controversial as well. Some authors reported a positive correlation between *p21^Cip1/Waf^* expression and favorable prognosis (174, 175), whereas others observed that *p21^Cip1/Waf^* expression is associated with poor survival (176).

Analysis of deletions and mutations of *p21^Cip1/Waf^* has been carried out in few human hematological malignancies and could be mapped in few subtypes. *p21^Cip1/Waf^* alterations are rare in typical mantle cell lymphoma (MCL), but loss of *p21^Cip1/Waf^* expression is present in aggressive MCLs harboring wild-type *p53* gene (177).

In a large cohort of AML patient blasts, high *p21^Cip1/Waf^* expression was found in AML1-ETO positive leukemia (178) with unknown significance. Given its role in maintaining the HSC-pool during normal hematopoiesis (37), one may speculate that it plays a role for LSCs by supporting their self-renewal capacity.

*p21^Cip1/Waf^* mutations appear to be not involved in childhood T-ALL pathogenesis, despite extensive studies no mutations were detected (179).

*p21^Cip1/Waf^* methylation status in leukemia still remains a debated topic. *p21^Cip1/Waf^* hypermethylation was observed in bone marrow cells derived from ALL patients, where it is indicative of a poor prognosis (180). Other studies failed to find any evidence for *p21^Cip1/Waf^* methylation in ALL and AML (155, 181, 182).

For instance, *p21^Cip1/Waf^* expression appears independent of its promoter methylation status in AML cell lines but correlates with demethylation of p73, a homologue of p53 and a known upstream transcriptional activator of *p21^Cip1/Waf^* (183). Treatment of AML cell lines with the methylation inhibitor 5-Aza-2′-deoxycytidine (5-Aza-CdR) results in the induced *p21^Cip1/Waf^* expression by p73 demethylation, provoking a cell cycle arrest in the G1 phase (184, 185). Decreased *p21^Cip1/Waf^* expression, without any signs of methylation, has been linked to higher disease aggressiveness in myelodysplastic syndrome (MDS). In line with the data from AML patients, reduced *p21^Cip1/Waf^* expression was commonly correlated to p73 methylation (186).

More studies are required to precisely understand how the *p21^Cip1/Waf^* methylation status interferes with disease progression and if p73 methylation can be used as a marker for the *p21^Cip1/Waf^* status.

In addition to growth arrest, p21^Cip1/Waf^ is involved in apoptosis, DNA repair and senescence. For instance, one of the most extensively studied functions of p21^Cip1/Waf^ is the protection of cells against apoptosis.

An example is given by the usage of histone deacetylase inhibitors (HDACI) to induce apoptosis (187–189). *p21^Cip1/Waf^* expression is upregulated by an increased histone acetylation of H3K4 at the *p21^Cip1/Waf^* promoter region, which is mediated by the HDACI SAHA (suberoylanilide hydroxamic acid) (190). *p21^Cip1/Waf^* overexpression confers resistance to SAHA-induced apoptosis which was shown in human AML cells. SAHA treatment promotes apoptotic cell death in leukemic cells by inducing pro-apoptotic genes such as TRAIL (TNF-related apoptosis-inducing ligand) and its downstream effector caspase-8. One mechanism through which p21^Cip1/Waf^ exerts anti-apoptotic effects in AML cell lines is the inhibition of caspase-8 cleavage to suppress TRAIL-mediated apoptosis (191).

A second anti-apoptotic function of p21^Cip1/Waf^ was also reported for AML blasts. There, high cytoplasmatic p21^Cip1/Waf^ protein levels provide protection against cytotoxic agents. Blasts with cytoplasmatic p21^Cip1/Waf^ levels show reduced etoposide (VP-16) mediated apoptosis (192). Similarly, the enforced expression of p21^Cip1/Waf^ in CML blast cells confers resistance to Imatinib induced apoptosis (193). These studies suggest that p21^Cip1/Waf^ expression should be investigated to act as a marker for therapeutic outcome.

*p21^Cip1/Waf^* expression is essential for the initiation and maintenance of leukemogenesis induced by PML/RAR-transformed HSCs. Under this condition p21^Cip1/Waf^ is required to maintain the self-renewal capacity of LSCs and to limit DNA-damage. p21^Cip1/Waf^ protects from functional exhaustion (194). In line p21^Cip1/Waf^ is crucial for the maintenance of self-renewal and chemoresistance of LSCs in a murine model of T-ALL (195).

In MLL-AF10-induced AML *p21^Cip1/Waf^* suppression is achieved by the oncomir miR-17-91, that is associated with enhanced LSC self-renewal and decreased leukemia latency (196). Functional studies for the role of p21^Cip1/Waf^ have been mainly carried out in cell lines from different leukemia subtypes. The literature on primary patient samples is scarce. It appears that the involvement of p21^Cip1/Waf^ is highly context dependent and relies on the differentiation status of the cells and on the driver oncogenes.

The fact that *p21^Cip1/Waf^* is important to maintain stem cell self-renewal might provide a basis for novel attempts to target p21^Cip1/Waf^ to induce exhaustion.

#### 5.2.2 p27^Kip1^

p27^Kip1^ regulates cell proliferation by inhibiting CDK complexes and arresting cell proliferation in response to anti-mitogenic signals (**Figure 1**) (8, 197–199).

Analysis of *p27^Kip1^* knock-out mice highlighted the importance of p27^Kip1^ as cell cycle regulator: *p27^Kip1^* deficient mice have an overall augmented cell proliferation which is reflected in increased body size and hyperplastic organs. Tumor formation becomes manifested spontaneously; pituitary and parathyroid tumors evolve and the mice show an increased susceptibility to tumorigenesis upon γ-irradiation or treatment by the chemical carcinogen N-ethyl-N-nitrosourea (ENU) (79, 80, 200). These studies defined p27^Kip1^ as tumor suppressor.

Mutations in the *p27^Kip^* ^1^ gene and its homozygous inactivation are generally rare in human cancers. In people *CDKN1B*, encoding for p27^Kip1^, has been identified as the second most common altered gene by frame-shift mutations in heterozygosity in hairy cell leukemia (HCL), a form of B-cell CLL. In most patients the *CDKN1B* mutation is clonal, thereby suggesting an early role in the pathogenesis of HCL (201, 202).

The subcellular location of p27^Kip1^ and its concentration determine the impact on malignant transformation. On the one hand, p27^Kip1^ acts as a tumor suppressor by inhibiting CDK-cyclin complexes and cell cycle progression when present in the nucleus. On the other hand, a localization shift of p27^Kip1^ from the nucleus to the cytoplasm, may promote tumor formation by regulating cytoskeletal structure and cell migration (89).

Augmented levels of p27^Kip1^ and its cytoplasmic localization have been correlated with poor prognosis and increased metastasis in diverse solid tumors including breast (94), cervix (97) and esophagus (95) carcinomas, as well as in some lymphoma and leukemia (91–93).

Despite a rare mutation rate, *p27^Kip1^* deregulation is one of the key events promoting leukemogenesis. Several mechanisms altering *p27^Kip1^* expression and localization have been described. miRNAs play a prominent role and abundance of p27^Kip1^ subjected to miRNA-mediated regulation: oncogenic expression of miRNA targeting *p27^Kip1^* translation can cause *p27^Kip1^* loss (203). In CML patients, increased miR-152-3p promotes aggressive behavior of CML cells by targeting p27^Kip1^ (204). Similarly, miR-148a correlates with low *p27^Kip1^* expression and increased proliferation in MM cells (205).

In lymphoma, low p27^Kip1^ levels correlate with a poor prognosis (206). Vice versa, high p27^Kip1^ levels are associated with enhanced disease-free survival in AML, indicative for disease progression (207).

In contrast, AML patients with low p27^Kip1^ due to deletion of the chromosomal region 12p13, have a better overall survival. Although together with *CDKN1B*, nine other genes are located in the 12p13 chromosomal region, the reported improved clinical outcome can be ascribed to reduced *CDKN1B* expression levels which might lead to higher cell proliferation which makes leukemic cells more susceptible to cytotoxic agents (208).

Besides the genomic alterations, also the phosphorylation sites play an important role for p27^Kip1^ levels. p27^Kip1^ is a substrate of FLT3 and FLT3-ITD in AML patient samples, where they phosphorylate p27^Kip1^ at the residue Y88 which is required for subsequent p27^Kip1^ phosphorylation at T187 by the CDK2-cyclin complex marking p27^Kip1^ for SCF^Skp2^-mediated degradation. FLT3 inhibition reduces pY88-p27^Kip1^ and increases p27^Kip1^ levels leading to cell cycle arrest (209).

High p27^Kip1^ levels are associated with a poor outcome in B-cell chronic lymphocytic leukemia (B-CLL). In B-CLL disease progression does not result from uncontrolled cell proliferation but is the result of defective apoptosis and enhanced cell survival. High *p27^Kip1^* expression is discussed to contribute to the protection against apoptotic stimuli like p21^Cip1/Waf^ (93).

The presence of high p27^Kip1^ levels in CLL was confirmed by others who also found an inverse correlation with c-Myc protein levels. C-Myc deregulation is a frequent event in leukemia and lymphoma (210, 211). Low Myc levels are associated with low expression of its target gene *Skp2*, a component of the SCF^Skp2^ ubiquitin ligase complex that degrades p27^Kip1^. The reduced Skp2-mediated degradation leads to the p27^Kip1^ accumulation which confers resistance to apoptosis (210).

In untransformed CD34^+^ progenitor cells, β_1_-integrin engagement increases p27^Kip1^ nuclear levels, which in turn decrease CDK2 activity thus restraining G1/S-phase progression. BCR-ABL expression in CML CD34^+^ cells induces elevated cytoplasmatic p27^Kip1^ levels. In this context, such high p27^Kip1^ levels do not restrain CML cell proliferation due to its cytoplasmatic relocation, thereby contributing to the loss of integrin-mediated proliferation inhibition observed in normal CD34^+^ cells (212).

More recent studies demonstrate that BCR-ABL1 promotes leukemia by subverting nuclear p27^Kip1^ tumor-suppressor function *via* two independent mechanisms. In a kinase-dependent manner, BCR-ABL1 induces SCF^Skp2^ expression through the PI3K pathway (213), promoting the degradation of nuclear p27^Kip1^, thus compromising its tumor-suppressor activity. In a kinase-independent fashion it increases cytoplasmatic p27^Kip1^ abundance, preventing apoptosis and thereby promoting leukemic cell survival (214, 215).

The overexpression of a stable p27^Kip1^ harboring two point mutations which prevent its phosphorylation on sites responsible for its SCF^Skp2^-mediated nuclear degradation (T187A) and for its PI3K-directed cytoplasmatic sequestration (T157A) causes a G1/S arrest, markedly inhibiting proliferation of BCR-ABL+ cells (216).

The complexity of the regulation mechanism regulation location and degradation require further investigations to define disease entities where p27^Kip1^ may serve as clinical marker.

#### 5.2.3 p57^Kip2^

Based on its ability to inhibit G1-S phase cyclin-CDK complexes, p57^Kip2^ is considered a tumor suppressor. As mentioned above for p21^Cip1/Waf^ and p27^Kip1^, p57^Kip2^ is involved in many cellular processes including apoptosis, and cellular migration.

The fact that p57^Kip2^ has a crucial role during embryogenesis and is required for normal embryonic development makes it unique under der CKI family. *p57^Kip2^* knock-out mice show severe developmental defects and display increased embryonic and perinatal lethality (217, 218) which complicated further studies on tumorigenesis in mice and most studies rely on human patient samples.

Reduced *p57^Kip2^* expression is associated with high tumor aggressiveness and poor prognosis in several types of tumors, such as gastric, colorectal, pancreatic, breast and lung carcinoma as well as leukemia (103, 104, 219–221). p57Kip2 expression is decreased in MDS, in particular in patients with a poor karyotype. Low expression results from an impaired response to the SDF-1/CXCR4 signal which induces *p57^Kip2^* expression (222). *p57^Kip2^* knock-out mice show hyperproliferation and differentiation delay in several tissues (218), which are features associated with the pathogenesis of MDS (223).

Another described mechanism how p57^Kip2^ expression is altered is promoter methylation. Hypermethylation of the *CDKN1C* gene, encoding for p57^Kip2^, occurs in diffuse large B-cell lymphoma (DLBCL), follicular lymphoma, ALL (224, 225) and nodal DLBCL (226). In the low-risk group of DLBCL, *CDKN1C* methylation is associated with a more favorable overall survival. The authors proposed aberrant *CDKN1C* promoter methylation as a biological marker in patients with DLBCL (226). Another study in DLBCL patients suggested that the analysis of *CDKN1C* methylation status may serve as a biomarker for the detection of minimal residual disease, underlining the importance of p57^Kip2^ for determining leukemia relapse risk (227).

Analysis of the *p57^Kip2^* methylation status in adult and childhood ALL found a rate of 50% *CDKN1C* hypermethylation in adult ALL but only 7% hypermethylation in childhood leukemia (226). Interestingly, in 53% of the childhood ALL samples p57^Kip2^ was absent without methylation and overall p57^Kip2^ levels were 8-fold lower compared to normal lymphocytes. The low expression points at additional ways to regulate *p57^Kip2^* in this particular disease class (228). *In line, p57^Kip2^ methylation and protein expression in adult ALL patients does not show any correlation* as 10 out of 15 patients with *CDKN1C* hypermethylation expressed p57^Kip2^ (229).

Overall, methylation status of *p57^Kip2^* does not seem to be a reliable marker for p57^Kip2^ levels. Conditional knockout mice would be a useful tool to study the role of p57^Kip2^ in hematopoietic diseases in more detail.

## 6 Pharmacologic CDK inhibition in hematologic malignancies

CDK kinase inhibitors are under extensive investigation in numerous preclinical and clinical studies in a variety of solid tumors and they are currently tested in hematological neoplasms (230, 231).

Pan-CDK inhibitors represented the very first generation of CDK inhibitors with the function to restrain cell proliferation *via* the inhibition of the CDK enzymatic activity. Flavopiridol was the first CDK inhibitor used in clinical trials and tested for the treatment of ALL, AML and CLL (232–234). Due to their low selectivity causing severe cytotoxic effects in healthy cells and a wide range of side effects, pan-CDK inhibitors have been discontinued in clinical trials (113, 235).

Considering the key role of CDK6 in malignant hematopoiesis it represents an effective therapeutic target (236–238). This is underlined by the high frequency of p15^INK4b^ and p16^INK4a^ inactivation in leukemia and lymphoma. The development of more specific CDK inhibitors, including CDK4/6-kinase inhibitors, represented an exciting turn over in the field (239).

Palbociclib is a CDK4/6 kinase inhibitor that acts by blocking enzymatic functions by mimicking INK4 binding. Palbociclib has been FDA approved to treat breast cancer patients and clinical trials exploring its effects in hematological malignancies are ongoing. Richter et al. present in their recent work (231) an extensive and detailed collection of preclinical and clinical studies conducted with several CDK4/6 inhibitors in hematological diseases.

Palbociclib resistance is a common phenomenon in breast cancer patients (240, 241). In breast cancer and AML high levels of p16^INK4a^ and p18^INK4c^ are associated with resistance to Palbociclib and to a CDK6 protein degrader that is based on the structure of Palbociclib. Despite this correlation, low p16^INK4a^ levels are not predictive for Palbociclib sensitivity (242). All INK4 proteins are in principle capable to prevent Palbociclib binding to CDK6 and thereby capable to induce resistance. Whether this fact is also true for other CDK inhibitors needs to be investigated. The cell-type specific expression of INK4 proteins needs also to be taken into consideration when studying CDK-inhibitors resistance.

The challenge in the development of novel inhibitors is in the design of molecules able to reduce the side effects and to overcome drug resistance. An innovative approach of CDK inhibition would consider the possibility to mimic the functions of INK4 proteins for a selective inactivation of CDKs. However, intensive research is needed to fill the need of X-ray crystal structures of most of the CDKs and CDKs/INK4/Cip/Kip complexes and to make this creative approach possible.

## 7 Discussion

INK4 and Cip/Kip proteins were initially identified as CDK inhibitors and negative regulators of cell cycle progression. Only recently, the involvement in other cellular processes including apoptosis and cell migration was uncovered. Thereby CKIs bridge cell cycle regulation to other cellular functions. Under certain circumstances CKIs may even promote cancer progression.

Tumor cells frequently display mutations in CKIs which underscores the significance of these proteins for tumorigenesis. We here summarize the dominant alterations of CKIs in hematopoietic malignancies and discuss their consequences for disease development, maintenance, and diagnosis.

Within the INK4 family, p15^INK4b^ and p16^INK4a^ are most frequently inactivated in leukemia and lymphoma either by deletion or hypermethylation of 5’ CpG islands in their promoter regions (114–116, 118, 140–150). The prognostic importance of these alterations in distinct disease entities remains unclear. Considering the unique functions of each INK4 proteins, especially their role under stress conditions, one could speculate that distinct expression patterns lead to different disease subtypes and dictates therapeutic outcomes.

CDK4/6 specific inhibitors represent a promising valuable choice for the treatment of hematological malignancies. However, resistance to CDK inhibitor therapy has been frequently observed. INK4 proteins are capable of inducing resistance by binding to CDK6. Studies are needed to evaluate whether this holds true for other CDK inhibitors.

As proliferation and cell cycle control are essential features of a cell, the components of the cell cycle machinery are present in multiple variants, which can substitute for each other. INK4 proteins share common tasks and, in a similar manner, CDKs may substitute for each other. This complexity makes it exceedingly difficult to generalize any consequence upon loss or mutations of a single player. Effects will also be context and cell type dependent.

This enormous plasticity of the cell cycle machinery to adapt ensures cell proliferation and presents a major challenge when it comes to predict therapeutic outcomes of drugs interfering with CDKs or INKs. The removal or inhibition of a single player may be rapidly compensated by a rearrangement of CDK complexes.

Another layer of complexity is induced by the emerging CDK6 kinase-independent functions that regulate transcriptional processes relevant for leukemia. The involvement of CDK6 in LSCs biology makes it an attractive target for leukemia therapy (238, 243). It is unclear how CKIs binding to CDK6 interferes with the transcriptional role of CDK6. It is also unknown whether INK4 or Cip/Kip binding to CDK6 alters the composition of CDK6 containing transcriptional complexes and/or chromatin location. We need to understand how CDK-CKIs complexes interfere with cell cycle-independent functions to reliable predict treatment outcomes. Moreover, effects of kinase inhibitor treatment on the kinase-independent functions of CDK6 are still enigmatic. The frequent upregulation of CDK6 (237, 235) in hematopoietic tumors (243, 244) and the fact that alterations of INK4 proteins are commonly found in hematopoietic tumors demands for the understanding of any CDK6-INK4 correlation in leukemia/lymphoma to exploit CDK4/6 inhibitors in hematopoietic malignancies.

Despite the importance of p18^INK4d^ for HSC self-renewal under homeostatic and stress conditions (40, 52,53), p18^INK4d^ mutations are not a hallmark of hematopoietic malignancies. p18^INK4d^ deregulation is rarely observed in hematopoietic neoplasms. Alterations on the transcriptional/translational level cannot be entirely excluded. As such the oncogene MLL-AF9 regulates p18^INK4d^. In line, the comparison of AML subtypes identified distinct INK4 expression patterns for different AML entities. The global analysis of the protein levels of individual CIKs in respect to their hematopoietic disease type is required to design tailored treatment strategies.

We are only starting to understand and appreciate functions of the Cip/Kip proteins in regulating apoptosis and cell migration. The involvement of Cip/Kip in tumorigenesis is an attractive emerging field of research and will open novel innovative therapeutic avenues.

p21^Cip1/Waf^ has a dual context-dependent role in leukemogenesis and acts as tumor suppressor and promoter. In cell lines, the anti-apoptotic effect of cytoplasmatic p21^Cip1/Waf^ confers a survival advantage and mediates chemoresistance. Inhibition of p21^Cip1/Waf^ under these conditions bears the potential to sensitize leukemic cells to chemotherapy. Similarly, cytoplasmatic p27^Kip1^ prevents apoptosis and may be exploited as potential therapeutic target. Most studies rely on cell lines and this only partially reflects the *in vivo* situation. The reality-check in patients is still missing to judge the clinical relevance of these observations. Therapeutic strategies that simultaneously target oncogenic Cip/Kip functions while preserving tumor suppressive functions would represent an innovative optimal approach.

## Author contributions

All authors made substantial, direct, and intellectual contributions to the work. KK was the principal investigator and takes primary responsibility for the paper. AS, VS and KK wrote the manuscript. All authors have read and agreed to the published version of the manuscript.

## Funding

Open Access Funding by the Austrian Science Fund (FWF), project grant P 31773 (KK), and the European Research Council under the European Union’s Horizon 2020 research and innovation programme, grant agreement no. 694354 (VS).

## Acknowledgments

Graphics were created with BioRender.com (24 March 2022). Open Access Funding by the University of Veterinary Medicine Vienna.

## Conflict of interest

The authors declare that the research was conducted in the absence of any commercial or financial relationships that could be construed as a potential conflict of interest.

## Publisher’s note

All claims expressed in this article are solely those of the authors and do not necessarily represent those of their affiliated organizations, or those of the publisher, the editors and the reviewers. Any product that may be evaluated in this article, or claim that may be made by its manufacturer, is not guaranteed or endorsed by the publisher.
